# A Comparative Analysis of Sonic Defences in Bombycoidea Caterpillars

**DOI:** 10.1038/srep31469

**Published:** 2016-08-11

**Authors:** Veronica L. Bura, Akito Y. Kawahara, Jayne E. Yack

**Affiliations:** 1 Department of Biology, Carleton University, 1125 Colonel By Drive, Ottawa, Ontario, K1S 5B6, Canada; 2 Florida Museum of Natural History, University of Florida, Gainesville, Florida, 32611, USA

## Abstract

Caterpillars have long been used as models for studying animal defence. Their impressive armour, including flamboyant warning colours, poisonous spines, irritating sprays, and mimicry of plant parts, snakes and bird droppings, has been extensively documented. But research has mainly focused on visual and chemical displays. Here we show that some caterpillars also exhibit sonic displays. During simulated attacks, 45% of 38 genera and 33% of 61 species of silk and hawkmoth caterpillars (Bombycoidea) produced sounds. Sonic caterpillars are found in many distantly-related groups of Bombycoidea, and have evolved four distinct sound types- clicks, chirps, whistles and vocalizations. We propose that different sounds convey different messages, with some designed to warn of a chemical defence and others, to startle predators. This research underscores the importance of exploring acoustic communication in juvenile insects, and provides a model system to explore how different signals have evolved to frighten, warn or even trick predators.

Acoustic communication in insects has been studied since antiquity, culminating in thousands of reports on diverse sounds, vibrations and sensory organs^1^,^2^,^3^. Most studies focus on adults, while reports on juveniles, such as caterpillars, grubs, maggots, nymphs and pupae, are conspicuously lacking. For example, defence sounds- produced when an insect is attacked or disturbed- are documented across many insect orders^4^, but juveniles are not included in reviews of this subject^4^,^5^,^6^. Yet arguably, immature insects such as caterpillars would benefit from evolving defence sounds, since they face severe predation by birds, shrews, mice, bats, frogs and lizards^7^,^8^ that have well developed hearing. Why then the dearth of evidence on defence sounds in juveniles? One possible explanation is that sound production is indeed rare- owing to the small sizes and soft body parts of most immature insects, sound production may not be an option. An alternative explanation is that sounds are prevalent, but have gone largely undetected by scientists.

Caterpillars of silk moths and relatives (Bombycoidea) are attacked by a wide variety of predators, and their visual and chemical defences are well-documented^7^,^9^,^10^,^11^,^12^. Despite the lack of evidence for caterpillar defence sounds historically, recent studies have shown that upon attack, some species of bombycids produce airborne sounds^13^,^14^,^15^,^16^. Sound producing species include the well-known tobacco hornworm (*Manduca sexta*), and Peacock moth (*Saturnia pyri*), that have been investigated for their antipredator behaviours, but for which defence sounds were not reported^17^,^18^. A comparative study of defensive sound production in bombycid larvae is therefore warranted to gain a better understanding of how sounds contribute to the defensive repertoires of larval insects. Moreover, Bombycoidea caterpillars can provide an excellent model system to test hypotheses on the functions of insect defence sounds^6^, because in caterpillars, sounds are less likely to serve overlapping functions in social or sexual contexts as they might in adults. This project had three goals: 1) to assess the prevalence of defence sounds among distantly-related Bombycoidea caterpillars across a wide geographic range; 2) to construct a multi-locus molecular phylogeny to explore how widespread such sounds are across the superfamily, and to establish whether different sound types are clade-specific; and 3) to conduct simulated attack trials on live caterpillars to test hypotheses on the functions of these sounds.

## Results and Discussion

**Figure 1:**
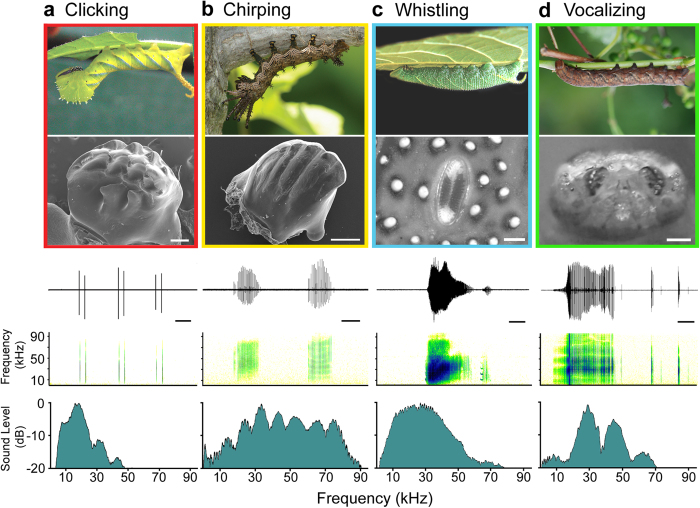
Moth caterpillars have evolved four different sonic defences. Sound producing mechanisms and corresponding acoustic characteristics in Bombycoidea caterpillars are illustrated by showing, from top to bottom, a representative species, the sound generating structure, a waveform and corresponding spectrogram of sound units (scale bars 100 ms), and a power spectrum. (**a**) Clicking. The death’s head hawkmoth caterpillar, *Acherontia atropos* (Sphingidae), produces short clicks using ridged ‘teeth’ on opposing mandibles. A single mandible is shown in the scanning electron micrograph (scale bar 250 μm), and three click units (each comprising two pulses in this case) are shown in the waveform. (**b**) Chirping. *Citheronia lobesis* (Saturniidae) creates chirps by sliding the serrated anterior edge of one mandible against the smooth inner surface of the opposing mandible. One mandible is shown in the scanning electron micrograph (scale bar 250 μm) and two chirp units in the waveform. (**c**) Whistling. The walnut sphinx caterpillar, *Amorpha juglandis* (Sphingidae), whistles by forcing air out of the 8th abdominal spiracles. A light micrograph of a single spiracle is shown (scale bar 250 μm), as well as a single whistle sound unit. (**d**) Vocalizing. The Nessus sphinx caterpillar, *Amphion floridensis* (Sphingidae), ‘vocalizes’ by forcing air out of its oral cavity. A light micrograph of opened mandibles (scale bar 1 mm) exposing the oral cavity during sound production is shown, and a waveform showing one long sound unit followed by two short ones.

Acoustic displays were made by 20/61 species (33%) and 17/38 genera (45%) of Bombycoidea that were studied. Sound-producing caterpillars occur in the wild silkmoths (Saturniidae), and hawkmoths (Sphingidae) in all geographic regions sampled (Table 1, Supplementary Table 1). Bombycoidea larvae make four distinct types of sounds: clicking, chirping, whistling and vocalizing (Fig. 1, Supplementary Movie 1). Clicks are produced by the mandibles, whereby the anterior edge of one mandible snaps against the serrated ridges on the inner face of the opposing mandible^13^,^16^ (Fig. 1a). Each movement produces one click unit (Fig. 1a). Click units on average are 16.1 ± 12.9 ms in duration with 2.1 ± 0.6 pulses (Table 1). Chirps are also produced by the mandibles, but differ from clicks in that the anterior edge of one mandible slides against the surface-textured inner face of the opposing mandible^14^ producing a train of pulses or ‘tooth-strikes’ (Fig. 1b). Chirp units are 70.4 ± 35.6 ms in duration with 11.7 ± 8.0 pulses (Table 1). Whistles are produced by air forced through the spiracles (breathing apparatus of insects)^15^ (Fig. 1c). Whistle units are 501.3 ± 100.5 ms in duration with 796.2 ± 382.1 pulses (Table 1). What we have named ‘vocalization’ is a previously unreported form of sound production for caterpillars. During sound production mandibles are held wide open, with air exiting the buccal cavity presumably through the foregut^19^ (Fig. 1d, Supplementary Movie 2). Vocalization units are 125.8 ± 81.3 ms in duration with 121.5 ± 186.9 pulses (Table 1). Each species examined produced only one type of sound. The diversity of sound-producing mechanisms in Bombycoidea caterpillars rivals that found within most groups of adult insects^20^. These results prompted us to ask the following questions: Are defence sounds and their respective mechanisms restricted to certain clades? Do different signal types have different functions?

**Figure 2:**
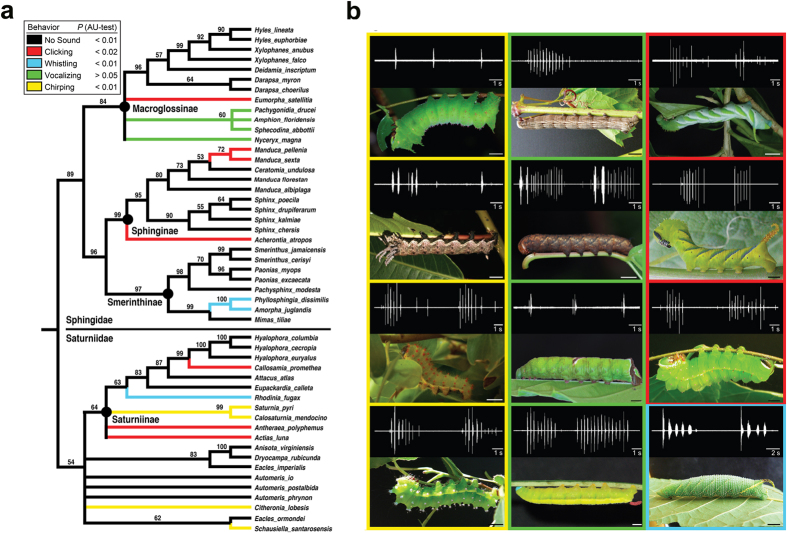
Evolution and diversity of defensive sounds in Bombycoidea caterpillars. (**a**) Phylogenetic relationships among the Sphingidae and Saturniidae species tested for defensive sound production. The phylogeny is the topology based on a maximum likelihood (ML) analysis; branch lengths and outgroups removed for visual simplicity (none of the outgroups produced sounds). Numbers above branches show bootstrap support values (>50%) from the ML analysis; branches with bootstrap ≤50% are collapsed (see Supplementary Fig. 1 for branch lengths). Ancestral state reconstruction was conducted in a multi-state parsimony mapping framework (5 states) on the ML tree. (**b**) Representative species are shown with a sound train. Chirping species (yellow borders) include from top to bottom, *Schausiella sanatarosensis*, *Citheronia lobesis*, *Calosaturnia mendocino* and *Saturnia pyri*. Vocalizing species (green borders) include from top to bottom, *Sphecodina abbottii*, *Amphion floridensis*, *Pachygonidia drucei* and *Nyceryx magna*. Clicking species (red borders) include, from top to bottom, *Manduca pellenia*, *Acherontia atropos* and *Actias luna*. One whistling species (blue border), *Amorpha juglandis*, is shown.

We examined our behavioural results on a 60-taxon, multi-locus maximum likelihood tree of Bombycoidea that we constructed. Ancestral states, inferring four different sounds – chirping, clicking, vocalizing, and whistling – were mapped onto this tree using a multi-state parsimony approach (Fig. 2, see Methods). Although phylogenies of bombycoids exist from previously published studies^21^,^22^,^23^,^24^,^25^,^26^, none of these studies include the taxon sampling that broadly captured both molecular sequence data and behavioural data to test the questions relevant for the present study. Our phylogeny is largely consistent with results from prior studies; relationships that were in conflict with some studies (e.g., *Manduca*^27^, Ceratocampinae, Attacini, Saturniini^23^,^25^,^28^) were not supported in our study with >65% bootstrap, and did not influence the overall conclusions on the evolution of sound production. Sound production is present in the Sphingidae and Saturniidae, occurring in five unrelated subfamilies: Ceratocampinae, Macroglossinae, Saturniinae, Smerinthinae, and Sphinginae (Table 1, Supplementary Table 1). Within a subfamily that includes sound-producing species, not all species tested produce sounds. Furthermore, three of the four sound-producing mechanisms - whistling, clicking and chirping - appear to have evolved multiple times, as they occur in different subfamilies and a single origin of each of these sounds is statistically rejected (Fig. 2). Whistling and clicking occur in both the Saturniidae and Sphingidae, chirping was found only in the Saturniidae, and vocalization only in Macroglossinae. Several congeners made the same sounds (Table 1, Supplementary Table 1) but other genera (*Citheronia, Manduca*) included both mute and sonic species. Only the Bombycidae and Hemileucinae lacked sound producing species among those tested. Outgroups examined did not produce sounds (Supplementary Table 1).

How might sounds protect caterpillars from predators? Sounds induced by a physical disturbance, variously named alarm, distress, warning or defence signals, are made by many insects, and range in complexity from those generated by non-specialized body parts (e.g. head banging) to those involving specialized sound producing organs (e.g. tymbals)^4^,^5^,^20^. Despite the diversity of defence sounds, their functions and the significance of their varied characteristics are poorly understood^6^. Sounds directed at predators are hypothesized to warn of a chemical or other defence (acoustic aposematism)^29^,^30^, advertize unprofitability, startle a predator (deimatic display)^29^, jam sonar signals^21^,^31^,^32^ or mimic another dangerous species^22^. Signals directed at conspecifics or heterospecifics may function to warn kin, or recruit help. Disentangling these hypotheses has been the subject of considerable and ongoing debate^6^. The rich diversity of acoustic displays in Bombycoidea caterpillars reported here makes them excellent subjects to test hypotheses on the functions of acoustic defence signals, since the confounding variables associated with adult reproductive functions are out of the picture.

**Figure 3:**
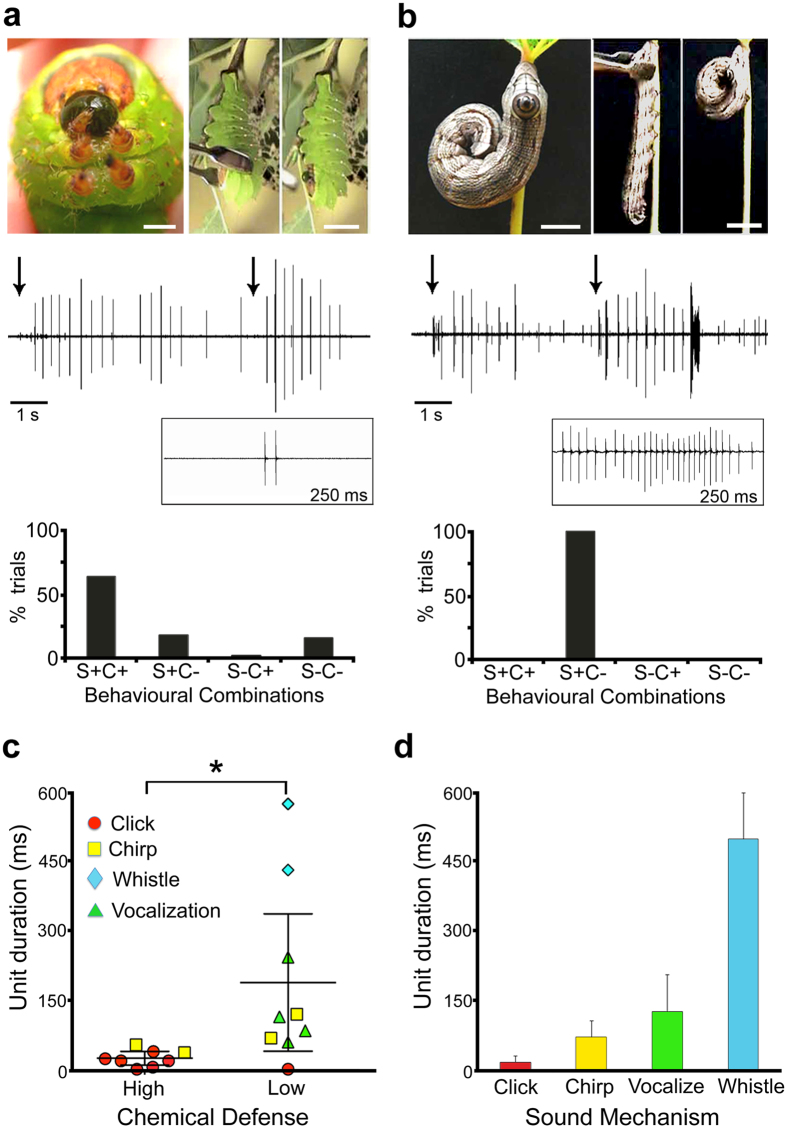
Different sound types may convey different messages to predators. (**a**) In some species, e.g., *Antheraea polyphemus* (Saturniidae), sound production is closely associated with a chemical defence, and proposed to function as a warning (aposematic) signal. Top panel: Photo on the left shows *A. polyphemus* regurgitating following sound production (scale bar: 2 mm). Right images are video snapshots of an individual being attacked with blunt forceps and subsequent regurgitation (scale bar: 10 mm). Middle panel: Sound waveform (10 s in duration) shows click trains following two successive attacks (arrows). The waveform in the box below is a 250 ms segment from the trace showing one expanded click unit comprising two pulses. Bottom panel: Temporal relationship between sound production and chemical release in five pinch trials showing that sound production typically precedes or accompanies the chemical defence (S+ Presence of sound; C+ Presence of chemical). (**b**) In other species e.g., *Sphecodina abbottii* (Sphingidae), sounds are not associated with a chemical defence and are proposed to function as a startle display. Top panel: Photo on the left shows *S. abbottii* displaying an eyespot while thrashing and vocalizing (scale bar: 2 mm). Right images are video snapshots before and after an attack with blunt forceps (scale bar: 10 mm). Middle panel: Sound waveform (10 s in duration) showing vocalization trains associated with two successive attacks (arrows). The waveform in the box below is a 250 ms segment from the trace showing one expanded vocalization. Bottom panel: No chemical defence was associated with five pinch attack trials. (**c**) Species that differ in their chemical score (high or low) also differ in their sound characteristics. Those with high chemical scores produce significantly shorter duration sounds, than species with low chemical scores (mean ± SEM) (Mann-Whitney U test, p ≤ 0.05). Low chemical species produce long duration (and higher energy) sounds, proposed to function in startle displays. (**d**) Sound mechanisms vary in their acoustic characteristics, such as unit duration. Sounds produced by clicking and chirping species tend to be shorter than those produced by whistling and vocalizing species (mean ± SD).

We propose that the caterpillar sounds recorded here are signals directed primarily at predaceous vertebrates. Three observations support this. First, a stimulus that evokes sound production is direct physical attack. During simulated attacks by a vertebrate predator with blunt forceps (see Methods), no sounds were recorded in the minute prior to attack, and 82%, 94%, and 95% of caterpillars generated sounds upon the first, second and third attacks respectively (N = 108 from 17 species). Following an attack, sound trains lasted between 0.2 to 32 s (Fig. 3a,b, Table 1, Supplementary Movie 1). Communication between conspecifics is unlikely, since late instar larvae of all the sound-producing species tested are typically solitary, and years of observations during rearing did not yield any evidence of sound production provoked by conspecific encounters. Second, an attacking vertebrate predator would hear these sounds – they are 2–570 ms in duration, have dominant frequencies between 15 and 42 kHz but are quite broadband (Table 1, Fig. 1), and range between 52–95 dB SPL at 5–10 cm^13^,^14^,^15^,^16^. These characteristics render them audible to common predators such as birds, (gleaning) bats, lizards and rodents^33^,^34^. Third, two species of birds- chickens, *Gallus gallus*, and yellow warblers, *Dendroica petechia*- respond to sound-producing caterpillars by stopping the attack, or flying away^13^,^15^. Caterpillar sounds may also affect invertebrate predators such as wasps, ants, beetles, true bugs and spiders^35^, but these predators typically lack tympanal ears^3^ and we assume that sounds would not usually be perceived by them or threatening to them. Still, there is the possibility that sounds are also transmitted as plant-borne vibrations and if so, may be accessible to invertebrates for purposes of recruiting help or defence.

What explains the diversity of caterpillar sounds? Insects use a variety of colour patterns to convey different messages- contrasting colours often signal toxicity, while eyespots frighten predators^9^,^10^,^36^. Likewise, different defence sounds may convey varied messages. We propose that in sonic caterpillars, some signals warn predators (acoustic aposematism), and others frighten them (startle displays). Acoustic aposematism typically occurs in species that use a chemical defence, and the sounds are predicted to precede or accompany chemical release to enhance the predator’s association with the chemical^6^,^30^,^37^. Startle sounds on the other hand are predicted to be unexpected and intense, and not directly associated with a chemical defence^6^. Eighteen species of sound-producing caterpillars, representing two families, five subfamilies, and 15 genera across the Bombycoidea (Table 1) were categorized into two groups based on the presence/absence and timing of chemical release during a series of sequential attacks (see Methods). In species with ‘high’ chemical scores, such as *Antheraea polyphemus* (Fig. 3a), the first sound produced precedes or accompanies the release of chemical from the mouth or scoli (specialized spines) in most (85.4%) trials (N = 100 individuals tested from nine species). These sounds are likely to inform the predator that the prey is unprofitable or unpleasant. Interestingly, all ‘high chemical’ species produced clicks or chirps, which have short durations (Fig. 3a,c,d; Table 1). Similar ‘click’ type sounds have been highly effective in operant conditioning paradigms to train vertebrates (e.g. clicker training^38^). Species with low chemical scores, such as *Sphecodina abbotti* (Fig. 3b), produce primarily whistles or vocalizations. Sounds produced by ‘low chemical’ species were significantly longer in duration (188.6 ± 64.0 ms, n = 9) than those of ‘high chemical’ species (25.7 ± 6.2 ms, n = 8) (Fig. 3c,d) (Mann-Whitney U test, p ≤ 0.05). We propose that these higher energy sounds have been selected to startle or frighten predators. Indeed, whistles produced by *Amorpha juglandis* caused yellow warblers (*Dendroica petechia*) to dive for safety^15^. Thus, short duration clicks and chirps are closely associated with chemical release, and we hypothesize are more effective in ‘educating’ predators, while longer duration, higher energy whistles and vocalizations lack an association with chemical release, and purportedly function as ‘acoustic eye spots’, to frighten predators by representing something dangerous. Similar results were reported for defence sounds in tiger moths (Erebidae: Arctiinae), whereby chemically defended species produced less sound per unit time than did undefended species^39^. Little is known about the significance of signal variation in arthropod defence sounds, and the subject offers fertile ground for future research^6^,^40^.

Despite centuries of research on insect acoustics, the topic of larval sound and vibration communication has been underrepresented in the literature^1^,^4^. Yet there is mounting evidence that immature insects engage in complex acoustic interactions within and between species e.g.^41^,^42^,^43^. We assert that scientists have barely scratched the surface on the topic of acoustic communication in juveniles, and the complexity and diversity of caterpillar defence sounds reported here underscores this assertion. Our study shows that among the species of Bombycoidea tested, one third are sonic. With an estimated 3,500 species occurring worldwide^44^ clearly much remains to be discovered. We hope that our results stimulate further exploration into the unchartered territories of larval sound and vibration communication, and acoustic warfare in arthropods.

## Methods

### Animals

Caterpillars used in this study were obtained from a variety of sources across Canada, U.S., Europe, and Costa Rica (Supplementary Table 1) over the course of the study period (2008–2014). All larvae used for experiments were late instars (penultimate and last). Bombycoidea species were selected based on their availability – any species available was tested – and information on defensive behaviours was obtained for a total of 61 species from 3 families and 8 subfamilies (Supplementary Table 1). Outgroup species used for behavioural studies included 18 species sampled across 5 superfamilies (Drepanoidea, Hesperioidea, Lasiocampoidea, Papilionoidea, Noctuoidea). The majority of specimens obtained from temperate regions were reared from eggs obtained from gravid females captured at ultraviolet and mercury vapor lights. A few individuals were collected as late instars from their respective host plants in the wild. Specimens were identified using standard field guides^7^,^45^. Temperate species were tested at Carleton University. Larvae from Costa Rica were collected as late instars from the wild at one of four sites in the Area de Concervación Guanacaste; Area Administrativa, Estacion San Gerardo, Estacion Caribe, and La Perla. All specimens collected in Costa Rica were tested on site. Voucher specimens are located at Carleton University, Ottawa. Information on sound production from two species, *Phyllosphinga dissimilis* and *Rhodinia fugax*, was obtained from other sources (Supplementary Table 1).

### Morphology

Caterpillars were photographed on their host plants using a Nikon D10 or Olympus SLR camera. Light micrographs of sound producing structures were taken with an Olympus SZX12 (Olympus Corporation, Tokyo, Japan) microscope equipped with an AxioCam MRc5 (Carl Zeiss Micro Imagin GmbH, Gottingen, Germany) camera. When possible, specimens were preserved in 80% ethanol and these specimens were used to examine mandibles. For scanning electron micrographs, mandibles were dissected from alcohol-preserved specimens, sputter-coated with gold-palladium and examined using a VEGA II XMU variable pressure SEM (Tescan USA Inc., Cranberry Twp., PA).

### Behavioural Trials

Individual late instar caterpillars were placed on sprigs of their host plant and isolated for 10–30 minutes prior to the beginning of the experiment. Simulated predator attacks^46^ were conducted by delivering multiple pinches to the head region using blunt forceps with ~5s between consecutive pinches. Behavioural and sound responses were recorded with a Sony handycam (DCR-HC85 or HDR-HC7, Tokyo, Japan) equipped with a Sony ECM microphone (ECM-MS907 or ECM-MS957, Tokyo, Japan) placed 2–5 cm away from the larva. Recordings at Carleton University were performed indoors in an enclosure lined with acoustic foam. Recordings in Costa Rica were performed outdoors at one of the research stations mentioned above, in a portable collapsible cage lined with acoustic foam. Trials were reviewed in Quicktime Pro 7.6.5 or iMovie 3.0.3 and analyzed for the occurrence of sound production and other defensive behaviours associated with each attack. Chemical defences associated with attack included regurgitation (from the oral cavity) or secretion of fluids from scoli (spine like protrusions from the body wall). These were categorized as ‘high’ or ‘low’ based on when the chemical appeared during the five pinch trials (adapted from Grant^46^: *High*. Chemical is secreted soon after attack (within the first 3 pinches on average), and the larva is able to control the release by reuptake, and direction of the attack. *Low*. Chemical is either not produced at all during the attack sequence, or if it is produced, it is not secreted until four or more attacks on average, and is thus considered a stress response). Mechanisms of sound production were individually assessed for each species by routinely videotaping body movements and close ups of mouthparts using a Sony HDR-HC7 HD Handycam (Tokyo, Japan) equipped with a Sony ECM-MS957 microphone and a macro lens^13^,^14^. Videos were analyzed using iMovie 3.0.3 to determine if and how mandible movements were associated with sound production.

### Sound recording and analysis

When a new sound-producing species was discovered, an additional set of recordings (in addition to those associated with video recordings) was conducted for analysis of sound characteristics. Individual caterpillars were stimulated to signal by delivering a pinch with blunt forceps as described above. Sounds were recorded with a Brüel & Kjær (Naerum, Denmark) 1/4” microphone (type 4939) (grid on) placed at determined distances (~5 or 10 cm from the sound producing structure), amplified with a Brüel & Kjær Nexus conditioning amplifier (type 2690), and recorded onto a Fostex FR-2 Field Memory Recorder (Gardena, CA, USA) at a sampling rate of 192 kHz. All recordings at Carleton University were performed in an acoustic chamber (Eckel Industries Ltd., Cambridge, MA, USA). In Costa Rica, specimens were brought indoors to a quiet room with minimal background noise and performed inside a portable acoustic enclosure lined with acoustic foam. Sounds were analyzed using either Raven Pro Bioacoustics Research Program 1.4 (Cornell Laboratory of Ornithology, Ithaca, NY, USA) or AviSoft SASlab Pro (Avisoft Bioacoustics, Berlin, Germany). Temporal characteristics measured included the train duration, sound unit duration, and number of pulses in a unit. A train is defined as a series of sound units following an attack until sound production ceases. We adopted the term unit to describe sounds that could be distinguished individually by the human ear in real time. Each sound unit is composed of one or more individual pulses (smallest divisible part of the waveform). Power spectra and spectrograms were produced using a 1024-point Fast Fourier Transform (Hann window, 50% overlap). Measured spectral characteristics included the dominant frequency and bandwidth at −10 dB from peak.

### Phylogenetic analysis and ancestral state reconstruction

A phylogenetic analysis was conducted on a dataset of 60 taxa, which were chosen based on the availability of behavioural and molecular data (Supplementary Tables 1, 2; Supplementary Fig. 1). A concatenated molecular dataset, which totaled 5789 nt, was assembled from the following five loci: the *pyrimidine biosynthesis* (*CAD*; 2931 nt)*, dopa decarboxylase* (*DDC*; 707 nt), *elongation factor-1α* (*ef-1α*; 1092 nt), *wingless* (402 nt), and a trimmed “barcode” region of *cytochrome oxidase 1* (*CO1*; 657 nt). The genetic data for the nuclear genes were assembled from previously available, published sequence datasets^21^,^23^,^24^,^25^ and CO1 barcode sequences were downloaded from the BOLD v3 online database ( www.boldsystems.org). We included Lasiocampidae because they represent important ancestral lineages within Bombycoidea^25^, and are outgroups to the Sphingidae and Saturniidae which are a sister family pair^26^,^47^,^48^, and the two focal families of the present study.

Individual-gene datasets were aligned using MAFFT 7.703^49^, implementing the E-INS-i option (mafft –genafpair maxiterate 1000). Alignments were visually inspected and checked for frame-shifts and the presence of termination codons with AliView 1.17^50^. Sequences were also assessed for contamination and sample-switching error by generating maximum likelihood (ML) bootstrap trees in RAxML 8.0^51^ for each gene. Each RAxML run consisted of a 1000-bootstrap analysis followed by a search for the best-scoring tree, incorporating the best scheme model obtained from PartitionFinder^52^. The final aligned data set was concatenated in FASconCAT 1.0^53^, and we implemented a topological constraint based on published trees^47^,^54^. We ran this ML constrained analysis in RAxML with 1000 ML tree searches and bootstraps on the University of Florida HPC cluster. The concatenated alignment, and scripts are deposited to the Dryad Data Repository ( http://www.datadryad.org).

Ancestral state reconstruction analyses were conducted in Mesquite 3.01^55^, on the topology of the most likely tree from the RAxML analysis. We rooted the tree with the ancestor of butterflies, based on the evidence that butterflies are outgroups to the Macroheterocera^47^,^48^,^56^,^57^,^58^ (Supplementary Fig. 1). We coded sound production as an unordered multi-state character with five states: 1) No sound; 2) Clicking; 3) Chirping; 4) Vocalizing; and 5) Whistling. We used multi-state parsimony ASR mapping using the “Trace Character History” option in Mesquite. Taxa for which we did not have behavioural data were coded as uncertain. We also conducted approximately unbiased tests (AU test^59^) in CONSEL 0.20^60^. The AU test determines if trees under a topological constraint describe the data significantly worse than the best (unconstrained) tree. The AU test was conducted to test whether the most parsimonious morphological inference (single origin of sound production for each sound type) was not attributable to sampling error in the molecular data. We built constrained trees in Mesquite and compared the statistical significance of these trees to the unconstrained ML tree in CONSEL. All GenBank accession numbers are listed in Supplementary Table 2. The dataset is also available via the Dryad Digital Repository ( www.datadryad.org).

## Additional Information

**How to cite this article**: Bura, V. L. *et al.* A Comparative Analysis of Sonic Defences in Bombycoidea Caterpillars. *Sci. Rep.* **6**, 31469; doi: 10.1038/srep31469 (2016).

## Supplementary Material

Supplementary Movie 1

Supplementary Movie 2

Supplementary Information

## Figures and Tables

**Table 1:** Acoustic and Chemical Defences in Sound Producing Bombycoidea Caterpillars.

Taxona,^b^	Acoustic Defence							Chemical Defence
	Sound type	Unit duration (ms)	Number of pulses/unit	Pulse rate^c^ (#/s)	Train duration^d^ (s)	Dominant frequency (kHz)	Bandwidth −10 dB from peak	Chemical score^e^
**SATURNIIDAE**								
Ceratocampinae								
* Citheronia lobesis*	Chirp	120.4 (42.9)	22.8 (89.2)	196.9 (57.0)	1.0 (0.3)	32.1 (10.8)	53.4 (22)	2
* Schausiella santarosensis*	Chirp	68.8 (16.8)	12.5 (1.2)	167.6 (9.7)	0.21 (0.3)	38.3 (1.6)	64.3 (24)	2
Saturniinae								
* Actias luna*	Click	40.1 (22.1)	2.4 (1.5)	147.1 (128.3)	5.4 (4.2)	26.5 (8.6)	25.7 (7.7)	1
Antheraea pernyi*	Click	N/A	N/A	N/A	N/A	N/A	N/A	1
* Antheraea polyphemus*	Click	20.0 (15.6)	3.2 (1.7)	200.5 (165.2)	8.5 (3.6)	15.9 (5.7)	19.8 (7.5)	1
* Antheraea polyphemus oculea*	Click	6.5 (10.3)	2.0 (1.1)	235.6 (130.1)	9.9 (6.2)	22.1 (7.3)	33.6 (6.1)	1
* Callosamia promethea*	Click	20.0 (7.4)	2.7 (0.6)	226.4 (156.1)	1.6 (0.3)	27.0 (2.3)	25.0 (3.6)	1
Rhodinia fugax*	Whistle	572.4 (40.7)	1066.4 (22.5)	1972.8 (301.6)	N/A	N/A	N/A	2
* Saturnia pyri*	Chirp	54.7 (21.3)	5.7 (2.1)	100.4 (12.1)	1.9 (0.9)	32.81 (6.0)	29.7 (0.5)	1
Calosaturnia mendocino*	Chirp	37.7 (21.2)	5.8 (1.8)	213.0 (97.6)	3.9 (1.8)	N/A	N/A	1
**SPHINGIDAE**								
Sphinginae								
Acherontia atropos*	Click	12.9 (6.7)	1.7 (0.8)	102.4 (25.7)	2.8 (1.4)	23.3 (3.0)	22.5 (7)	N/A
* Manduca pellenia*	Click	2.2 (4.5)	1.3 (0.5)	158 (138.7)	2.8 (1)	17.6 (17)	18.43 (13)	1
* Manduca sexta*	Click	24.7 (27.5)	2.2 (1.4)	98.4 (53.6)	3.4 (2.9)	27.6 (11.0)	23.8 (4)	1
Smerinthinae								
* Amorpha juglandis*	Whistle	430.4 (272)	526.0 (121.4)	2301 (95.9)	3.8 (2)	15.3 (4)	6.7 (1.4)	2
Phyllosphingia dissimilis*	Whistle	N/A	N/A	N/A	N/A	N/A	N/A	N/A
Macroglossinae								
* Amphion floridensis*	Vocalize	243.1 (108.2)	401 (192)	1190.8 (300.1)	32.3 (29.1)	40.8 (2.9)	37.1 (6.2)	2
Eumorpha satellitia*	Click	2.2 (2.1)	1.8 (0.5)	N/A	0.7 (0.4)	N/A	N/A	2
* Nyceryx magna*	Vocalize	60.5 (11.7)	13.9 (3.4)	289 (81.5)	4.5 (4.1)	24.8 (13)	26.7 (13)	2
* Pachygonidia drucei*	Vocalize	84.8 (61.9)	50.5 (8.5)	390.6 (149.5)	0.3 (0.1)	42.9 (11)	64.0 (7)	2
* Sphecodina abbottii*	Vocalize	114.7 (21)	20.8 (11.9)	227.7 (68.5)	7.1 (4.4)	29.0 (14.5)	27.0 (1.5)	2

^a^“Taxa names were obtained from the Natural History Museum, London, U.K. Lepindex website. Beccaloni, G. *et al.*, *Natural History Museum - The Global Lepidoptera Names Index.* (2005) Available at: http://www.nhm.ac.uk/our-science/data/lepindex/. (Accessed: 15th July 2015)”.

^b^Values reported in this table were obtained from 3 unit measurements from each of 5 individuals (or fewer if 5 were not available). The total number of specimens tested for defensive behaviours is listed in Supplementary Table 1. Values expressed as Mean (SD).

^c^Pulse rates were obtained only from units that had 2 or more pulses.

^d^Duration of sound train (series of units) following attack.

^e^Chemical score of 1 is high and score of 2 is low (see Methods).

^*^Incomplete information for these species. *A. pernyi* was tested for sound production but sound and video clips were not obtained. *P. dissimilis* information was obtained from the literature. *R. fugax* information was obtained from video recordings only. Spectral information was not available for *C. mendocino* and *E. satellitia* due to the nature of the recordings. Sound but not video recordings were obtained for *A. atropos*. See Supplementary Table 1 for details.
