# Supplementary material for: A Comparative Analysis of Sonic Defences in Bombycoidea Caterpillars
Source: Sci Rep. 2016 Aug 11;6:31469. doi: 10.1038/srep31469 (PMC4980592; doi:10.1038/srep31469)
Supplement: Supplementary Information [file srep31469-s3.pdf]

A Comparative Analysis of Sonic Defences in Bombycoidea Caterpillars

Veronica L. Bura[1], Akito Y. Kawahara[2], Jayne E. Yack[1*]

[1]Department of Biology, Carleton University, 1125 Colonel By Drive, Ottawa, Ontario, K1S 5B6, Canada
[2]Florida Museum of Natural History, University of Florida, Gainesville, Florida, 32611, USA

[*]Corresponding author: Dr. Jayne E. Yack, Department of Biology, Carleton University, 1125 Colonel By Drive, Ottawa, ON, Canada, K1S 5B6; Email: jayneyack@cunet.carleton.ca; Tel: 613 520 2600 (Ext. 3887)

Supplementary Table 1
Summary of all species examined for defensive behaviours between 2005-2014.

Taxonomic Group[a]			Collection Data			Sound Production	
**Family**	**Subfamily**	**Species**	**No. of specimens (No. of broods)**	**Food plant**	**Collection location**	**Y/N**	**Type**
Bombycidae	Bombycinae	*Bombyx mori* Linnaeus	10	*Morus rubra*	Ottawa, ON (Pet store)	No	n/a
	Apatelodinae	*Hygrochroa torrefacta* Smith	28	*Prunus* sp.	Arizona	No	n/a
		Olceclostera angelica (Grote)	50+ (2)	*Fraxinus* sp., *Syringa* sp.	Chaffey's Lock (QUBS), ON	No	n/a
Saturniidae	Ceratocampinae	*Anisota virginiensis* (Drury)	70+ (2)	*Quercus* sp.	Chaffey's Lock (QUBS), ON	No	n/a
		Citheronia lobesis Rothschild	2	*Cochlospermum vitifolium*	Costa Rica, Guanacaste	Yes	Chirping
		Citheronia splendens (Druce)	50+	*Rhus* sp.	Arizona	No	n/a
		Dryocampa rubicunda (Fabricius)	85+ (2)	*Acer saccharum*	Ottawa (Mer Bleue), ON & PEI	No	n/a
		Eacles imperialis (Drury)	50+ (2)	*Pinus* sp., *Picea* sp.	Chaffey's Lock (QUBS), ON	No	n/a
		Eacles ormondei Schaus	1	*Tapirira mexicana*	Costa Rica	No	n/a
		Eacles oslari Rothschild	5	*Quercus alba*	Arizona	No	n/a
		Schausiella santarosensis Lemaire	3	*Hymenaea courbaril*	Costa Rica	Yes	Chirping
	Hemilucinae	*Automeris postalbida* Schaus	1	*Acalypha diversifolia*	Costa Rica	No	n/a
		Automeris io (Fabricius)	80+ (3)	*Betula papyrifer*, *Acer* sp., *Populus* sp.	Chaffey's Lock (QUBS), ON & PEI	No	n/a
		Automeris phrynon Druce	1	*Terminalia catappa*	Costa Rica	No	n/a
		Automeris cecrops pamina Neumoegen	10+	*Quercus* sp.	Arizona	No	n/a
	Saturniinae	*Actias luna*	100+ (3)	*Betula papyrifera*	Chaffey's Lock	Yes	Clicking

		(Linnaeus)			(QUBS), & Ottawa (Mer Bleue), ON		
		Antheraea pernyi (Guérin-Méneville)	2	*Quercus* sp.	Italy	Yes	Clicking
		Antheraea polyphemus (Cramer)	200+ (3)	*Quercus rubra, Betula papyrifera, Acer saccharum*	Chaffey's Lock (QUBS), & Ottawa (Mer Bleue), ON & PEI & Massachusetts	Yes	Clicking
		Antheraea polyphemus oculea (Neumoegen)	45	*Quercus* sp.	Arizona	Yes	Clicking
		Attacus atlas (Linnaeus)	8	*Citrus* sp.	South East Asia	No	n/a
		Callosamia promethea (Drury)	34 (3)	*Betula* sp.	Chaffey's Lock (QUBS), ON & PEI	Yes	Clicking
		Calosaturnia mendocino (Behrens)	4	*Arctostaphylos* sp. or *Arbutus menziesii*	California	Yes	Chirping
		Eupackardia calleta (Westwood)	2	*Prunus domestica*	Arizona	No	n/a
		Hyalophora cecropia (Linnaeus)	20+	*Betula papyrifera*	Chaffey's Lock (QUBS)	No	n/a
		Hyalophora columbia (SI Smith)	20+	*Betula* sp.	Arizona	No	n/a
		Hyalophora columbia gloveri (Strecker)	6	*Prunus domestica*	Arizona	No	n/a
		Hyalophora euryalus (Boisduval)	2	*Prunus* sp.	Arizona	No	n/a
		*Rhodinia fugax** (Butler)	n/a	n/a	n/a	Yes	Whistle
		Saturnia pyri (Denis & Schiffermüller)	52 (2)	*Populus* sp.	Denmark & Switzerland	Yes	Chirping
Sphingidae	Sphinginae	*Acherontia atropos* (Linnaeus)	5	*Solanum* sp.	Africa	Yes	Clicking
		Ceratomia undulosa (Walker)	20	*Fraxinus* sp.	Chaffey's Lock (QUBS), ON	No	n/a
		Manduca albiplaga (Walker)	1	*Annona rensoniana*	Costa Rica	No	n/a
		Manduca florestan (Stoll)	1	plant 19080 *(Bignoniaceae)*	Costa Rica	No	n/a
		Manduca pellenia (Herrich-Schaffer)	1	*Cestrum megalophyllum*	Costa Rica	Yes	Clicking
		Manduca sexta (Linnaeus)	60+ (3)	*Solanum lycopersicum, Datura inoxia, Nicotiana tabacum,* Artificial diet	Dalhousie University, NS & Mercier (supplier), QC & Windsor (supplier), ON	Yes	Clicking
		Sphinx chersis (Hubner)	1	*Fraxinus* sp.	Ottawa, ON	No	n/a
		Sphinx drupiferarum Abbot & Smith	16	*Prunus* sp. (plum)	PEI	No	n/a
		Sphinx kalmiae Abbot & Smith	20	*Fraxinus* sp.	PEI	No	n/a

		Sphinx poecila Stephens	18	*Vaccinium* sp.	PEI	No	n/a
	Smerinthinae	*Adhemarius ypsilon* (Rothschild & Jordan)	1	*Ocotea atirrensis*	Costa Rica	No	n/a
		Amorpha juglandis (Abbot & Smith)	18 (2)	*Ostyra* sp., *Alnus* sp., *Juglans* sp., *Fagus* sp.	Chaffey's Lock (QUBS), ON	Yes	Whistle
		Mimas tiliae (Linnaeus)	3	*Alnus* sp.	Europe	No	n/a
		Pachysphinx modesta (Harris)	8	*Populus tremuloides*	PEI	No	n/a
		Paonias excaecata (Abbot & Smith)	14 (2)	*Betula* sp.	Chaffey's Lock (QUBS), ON & PEI	No	n/a
		Paonias myops (Abbot & Smith)	20	*Prunus* sp. (cherry)	Chaffey's Lock (QUBS), ON	No	n/a
		*Phyllosphingia dissimilis** (Bremer)	n/a	n/a	n/a	Yes	Whistle
		Smerinthus cerisyi Kirby	10	*Populus tremuloides*	Ottawa (Mer Bleue), ON	No	n/a
		Smerinthus jamaicensis (Drury)	5	*Populus tremuloides*	Ottawa (Mer Bleue), ON	No	n/a
	Macroglossinae	*Amphion floridensis* Clark	130+ (2)	*Vitis* sp.	Florida	Yes	Vocalization
		Darapsa choerilus (Cramer)	5	*Viburnum* sp.	PEI	No	n/a
		Darapsa myron (Cramer)	50+ (2)	*Vitis* sp.	Ottawa, ON & Chaffey's Lock (QUBS), ON	No	n/a
		Deidamia inscriptum (Harris)	1	*Vitis* sp.	Chaffey's Lock (QUBS), ON	No	n/a
		Eumorpha satellitia (Linnaeus)	2	*Vitis tiliifolia*	Costa Rica	Yes	Clicking
		Hyles euphorbiae (Linnaeus)	3	*Euphorbia* sp.	Ottawa	No	n/a
		Hyles lineata (Fabricius)	5	*Oenothera* sp.	Arizona	No	n/a
		Nyceryx magna (Felder)	1	*Pentagonia donnell-smithii*	Costa Rica	Yes	Vocalization
		Pachygonidia drucei (Rothschild & Jordan)	1	*Doliocarpus multiflorus*	Costa Rica	Yes	Vocalization
		Sphecodina abbottii (Swainson)	3	*Vitis* sp.	Ottawa, ON	Yes	Vocalization
		Xylophanes anubus (Cramer)	1	*Psychotria panamensis*	Costa Rica	No	n/a
		Xylophanes tyndarus (Boisduval)	1	*Faramea occidentalis*	Costa Rica	No	n/a
		Xylophanes falco (Walker)	4	*Bouvardia glaberrima*	Arizona	No	n/a
**OUTGROUPS**							
Lasiocampidae	Macromphaliinae	*Euglyphis amathuria*DHJ02	1	*Cinnamomum brenesii*	Costa Rica	No	n/a

Noctuidae	Catocalinae	*Acanthodica sinuilinea*DHJ02	1	*Heteropterys obovata*	Costa Rica	No	n/a
Notodontidae	Dudusinae	*Crinodes besckei*	1	*Gouania* sp.	Costa Rica	No	n/a
	Dicranurinae	*Lirimiris fascis*	1	*Mortoniodendron guatemalense*	Costa Rica	No	n/a
		Lirimiris guatemalensis	1	*Guazuma ulmifolia*	Costa Rica	No	n/a
Nymphalidae	Morphinae	*Antirrhea lindigii* Felder	1	*Geonoma ferruginea*	Costa Rica	No	n/a
		Morpho amathonte Deyrolle	1	*Lonchocarpus oliganthus*	Costa Rica	No	n/a
	Nymphalinae	*Smyrna blomfildia* (Fabricius)	1	*Urera caracasana*	Costa Rica	No	n/a
	Danainae	*Danaus* sp.	1	*Solandra grandiflora*	Costa Rica	No	n/a
Hesperiidae	Eudaminae	*Astraptes creteus crana* Evans	1	*Styrax argenteus*	Costa Rica	No	n/a

[a] "Taxa names were obtained from the Natural History Museum, London, U.K. Lepindex website. Beccaloni, G. et al., *Natural History Museum - The Global Lepidoptera Names Index*. (2005) Available at: http://www.nhm.ac.uk/our-science/data/lepindex/. (Accessed: 15th July 2015)"
* Information on *Rhodinia fugax* is from the literature and non-lab created videos. Information on *Phyllosphingia dissimilis* is from the literature only.

Supplementary Table 2
Taxa and their GenBank accession numbers for each locus used to construct the ML tree in this study.

Family	Genus/species	CAD	COI	DDC	EF1	WG
Saturniidae	*Actias_luna*	*	GU087182	*	AF015069	*
Saturniidae	*Anisota_virginiensis*	*	GU087657	*	*	*
Saturniidae	*Antheraea_pernyi*	*	EU532613	AY461438	FJ788508	*
Saturniidae	*Antheraea_polyphemus*	EU032645	GU438053	AF373953	AF373927	EU033059
Saturniidae	*Attacus_atlas*	EU032631	AB533579	EU032768	AF015066	EU033049
Saturniidae	*Automeris_io*	EU032648	GU088608	EU032784	*	EU033062
Saturniidae	*Automeris_phrynon*	*	GU146236	*	*	*
Saturniidae	*Automeris_postalbida*	*	JQ529319	*	*	*
Saturniidae	*Callosamia_promethea*	*	*	AF015052	AF015073	*
Saturniidae	*Ceratomia_undulosa*	*	GU438133	*	*	*
Saturniidae	*Citheronia_lobesis*	*	JQ570223	*	*	*
Saturniidae	*Dryocampa_rubicunda*	*	GU087259	*	AF234564	*
Saturniidae	*Eacles_ormondei*	*	JQ579310	*	*	*
Saturniidae	*Eupackardia_calleta*	*	*	AF015054	AF015075	*
Saturniidae	*Hyalophora_cecropia*	EU032685	KM287185	EU032818	AF015077	EU033090
Saturniidae	*Hyalophora_columbia*	*	KM287192	*	*	*
Saturniidae	*Hyalophora_euryalus*	EU032687	HM867376	AF015057	AF015078	*
Saturniidae	*Rhodinia_fugax*	*	GU663933	AF015061	*	*
Saturniidae	*Calosaturnia_mendocino*	*	*	EU032878	AF373945	EU033146
Saturniidae	*Saturnia_pyri*	*	JN278459	*	DQ077816	*
Saturniidae	*Schausiella_santarosensis*	*	DQ266740	*	*	*
Sphingidae	*Acherontia_atropos*	EU479012		EU479122	EU479232	EU479446
Sphingidae	*Amorpha_juglandis*	EU479021	GU088582	EU479131	EU479241	EU479455
Sphingidae	*Amphion_floridensis*	*	JN677742	*	EU479244	EU479458
Sphingidae	*Darapsa_myron*	EU479045	KP720048	EU479155	EU479266	EU479480
Sphingidae	*Deidamia_inscriptum*	EU479046	KP720047	EU479156	*	EU479481
Sphingidae	*Eumorpha_satellitia*	*	HQ581557	*	KP720269	KM456038
Sphingidae	*Hyles_euphorbiae*	*	HQ955341	*	*	FN393414

Sphingidae	*Hyles_lineata*	EU032688	JN678058	EU479176	EU479288	EU033092
Sphingidae	*Manduca_albiplaga*	KC893229	GU152246	*	KC893193	KC893213
Sphingidae	*Manduca_florestan*	*	JQ572523	EU479186	EU479298	EU479511
Sphingidae	*Manduca_pellenia*	KC893240	DQ276326	*	KC893201	KC893216
Sphingidae	*Manduca_sexta*	EU032707	HQ581428	U03909	AF234571	EU033109
Sphingidae	*Mimas_tiliae*	EU479079	KP720098	EU479192	EU479304	EU479517
Sphingidae	*Nyceryx_magna*	*	DQ276382	EU479196	EU479310	EU479523
Sphingidae	*Pachygonidia_drucei*	*	DQ276419	*	*	*
Sphingidae	*Pachysphinx_modesta*	*	JN678337	*	AF234573	*
Sphingidae	*Paonias_excaecata*	*	GU090083	*	AF234572	*
Sphingidae	*Paonias_myops*	*	GU091309	EU479202	AF234574	EU033131
Sphingidae	*Phyllosphingia_dissimilis*	EU479095	JN678367	EU479207	EU479320	EU479533
Sphingidae	*Smerinthus_cerisyi*	*	HM862530	AF234595	AF234576	*
Sphingidae	*Smerinthus_jamaicensis*	*	JN678502	*	*	*
Sphingidae	*Sphecodina_abbottii*	EU479106	GU089266	EU479218	AF234575	EU479544
Sphingidae	*Sphinx_chersis*	*	GU438647	AF234596	AF234577	*
Sphingidae	*Sphinx_drupiferarum*	*	HM867045	*	*	*
Sphingidae	*Sphinx_kalmiae*	EU479111	GU091429	EU479223	EU479336	EU479549
Sphingidae	*Sphinx_poecila*	*	GU092657	*	*	*
Sphingidae	*Xylophanes_anubus*	KP719988	DQ276577	*	*	KP720004
Sphingidae	*Xylophanes_falco*	*	*	KP720009	AF234580	*
Apatelodidae	*Apatelodes_torrefacta*	EU032647	GU089655	EU032783	*	EU033061
Bombycidae	*Bombyx_mori*	EU032656	AB649182	AF372836	NM001044045	EU033069
Erebinae	*Acanthodica_sinuilinea*	*	JQ523982	*	*	*
Hesperiidae	*Astraptes_creteus crana*	*	JF777604	*	*	*
Notodontidae	*Lirimiris_guatemalensis*	JQ784619	GU158132	*	*	JQ786889
Notodontidae	*Crinodes_besckei*	JQ784377	GU332885	JQ785738	*	JQ786709
Nymphalidae	*Smyrna_blomfildia*	*	JQ548137	*	AY788816	AY788576
Nymphalidae	*Danaus_plexippus*	*	GU659709	*	KJ496142	JQ786769
Nymphalidae	*Antirrhea_lindigii*	*	GU156905	*	*	*
Nymphalidae	*Morpho_amathonte*	*	JQ536411	*	*	JN696117

Supplementary Figure 1: Supplementary Figure x. RAxML maximum likelihood tree showing branch lengths and outgroups. Bootstrap values >50% are shown above branches.

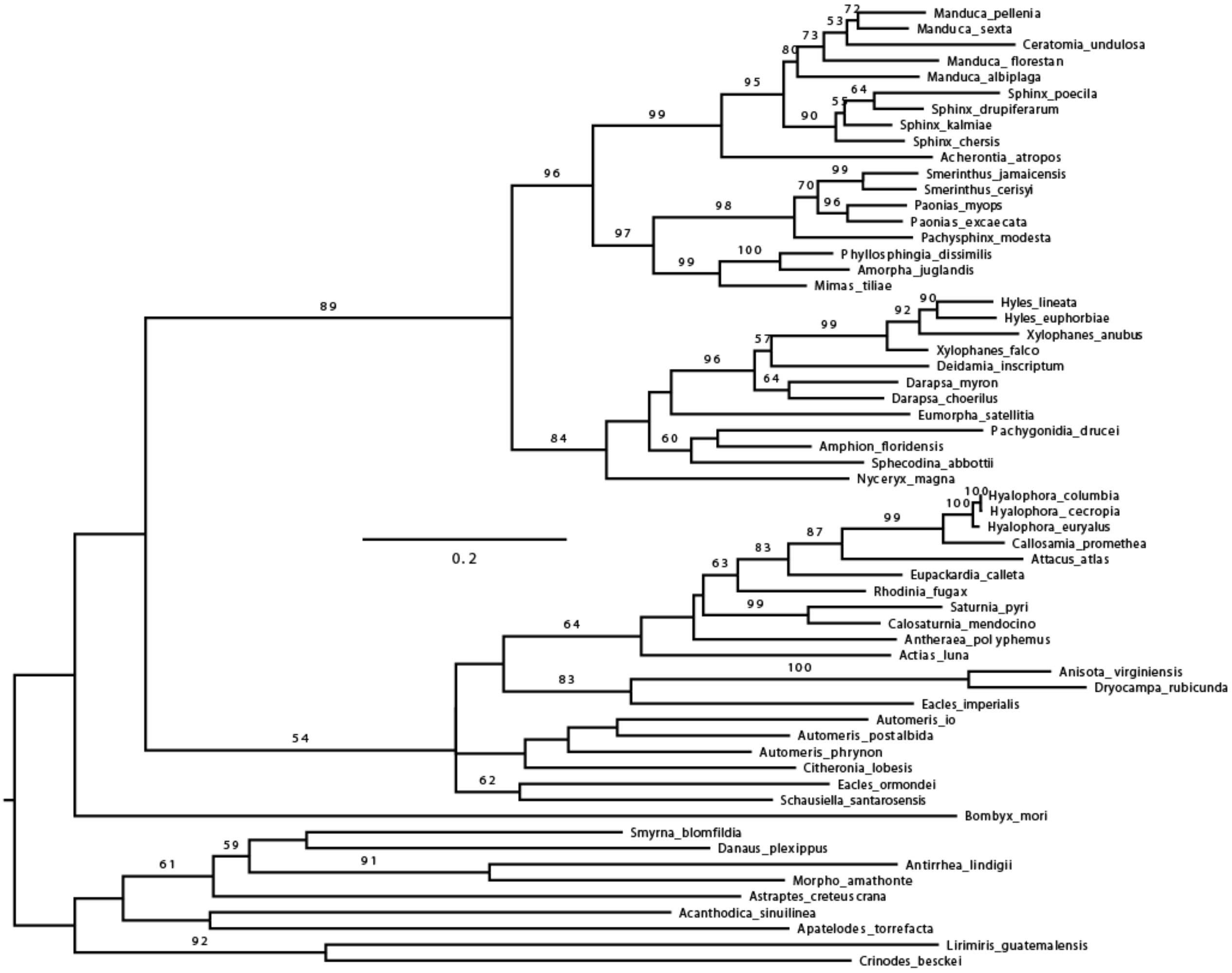

Supplementary Movie 1 (Caption)
Defensive sounds in caterpillars belonging to the silk and hawk moths (Bombycoidea). Videoclips show four different sound producing mechanisms (clicking, chirping, whistling and vocalizing) following simulated predator attacks.

Supplementary Movie 2 (Caption)
Close up video of mouthparts during 'vocalization' sound production in the Abbott's sphinx (*Sphecodina abbottii*). The first part of the video is played in real time, and the second part is slowed down to show how mouth-parts are held wide open during sound production.
